# Physiological Effects of Touching Wood

**DOI:** 10.3390/ijerph14070801

**Published:** 2017-07-18

**Authors:** Harumi Ikei, Chorong Song, Yoshifumi Miyazaki

**Affiliations:** 1 Department of Wood Engineering, Forestry and Forest Products Research Institute, 1 Matsunosato, Tsukuba, Ibaraki 305-8687, Japan; ikei0224@ffpri.affrc.go.jp; 2 Center for Environment, Health and Field Sciences, Chiba University, 6-2-1 Kashiwa-no-ha, Kashiwa, Chiba 277-0882, Japan; crsong1028@chiba-u.jp

**Keywords:** wood, tactile, autonomic nervous activity, prefrontal cortex activity, heart rate variability, near-infrared spectroscopy, semantic differential method, profile of mood states, physiological relaxation, preventive medical effect

## Abstract

This study aimed to clarify the physiological effects of touching wood with the palm, in comparison with touching other materials on brain activity and autonomic nervous activity. Eighteen female university students (mean age, 21.7  ±  1.6 years) participated in the study. As an indicator of brain activity, oxyhemoglobin (oxy-Hb) concentrations were measured in the left/right prefrontal cortex using near-infrared time-resolved spectroscopy. Heart rate variability (HRV) was used as an indicator of autonomic nervous activity. The high-frequency (HF) component of HRV, which reflected parasympathetic nervous activity, and the low-frequency (LF)/HF ratio, which reflected sympathetic nervous activity, were measured. Plates of uncoated white oak, marble, tile, and stainless steel were used as tactile stimuli. After sitting at rest with their eyes closed, participants touched the materials for 90 s. As a result, tactile stimulation with white oak significantly (1) decreased the oxy-Hb concentration in the left/right prefrontal cortex relative to marble, tile, and stainless steel and (2) increased ln(HF)-reflected parasympathetic nervous activity relative to marble and stainless steel. In conclusion, our study revealed that touching wood with the palm calms prefrontal cortex activity and induces parasympathetic nervous activity more than other materials, thereby inducing physiological relaxation.

## 1. Introduction

Wood is a familiar natural material that has been used in houses and furniture for a long time, and it is empirically known to have a relaxing effect on humans. In Japan, a majority (55%) of new housing starts in 2014 are wooden, and among them, the percentage of wooden houses in detached houses is 88%, which is high [1]. According to an “awareness and intention survey on circulation utilization of forest resources” conducted by the Ministry of Agriculture, Forestry and Fisheries in 2015 [2], approximately 80% of the respondents answered “wooden houses” to the question of “a house you would like to choose in the future when building or buying houses”. In recent years, the Forestry Agency has been encouraging the use of wood and is expanding its *moku-iku* (“moku” implies “wood” and “iku” implies “nurture”) initiative [1]. First coined in 2004 [2], *moku-iku* is an expression that has been defined in many ways; these include “an initiative to encourage all people, including children, to interact with wood, learn from wood, and live with wood (Wood Culture Promotion Project Team [2])” and “educational activities regarding the use of wood to teach about the merit of wood as a material and the significance of using wood (Forestry Agency [1])”. Since then, Miyazaki has proposed a new concept of *moku-iku*: how the “quality of life is improved by being brought up in the presence of wood” and that “contact with wood is physiologically relaxing and enhances immune function” [3].

As described above, the interest in and expectations of the relaxing effect of wood on humans have increased in recent years, and data based on scientific evidence is awaited. The authors conducted a literature search to outline the current state of research regarding the physiological effects of wood-derived stimulation on humans [4]. The oldest report, published in 1992 [5], is on the physiological effect of Taiwan cypress oil by olfactory stimulation in human subjects, and albeit scarce, the reports have continued to accumulate until today, following the development of physiological measurement technology in recent years [4]. However, most of the previous studies about the physiological effects of wood or wood-derived stimulation on humans have used olfactory stimulation, and there are extremely few reports on tactile stimuli. Morikawa et al. [6] reported that touching artificial materials with the palm resulted in great fluctuations in the systolic blood pressure and pulse rate and induced a physiological stress state, whereas touching Japanese cypress and Japanese cedar wood plates caused little fluctuation. Sakuragawa et al. [7] examined differences in the effects of tactile stimulation on human physiology that resulted from materials at different temperatures (cool, room temperature, and warm). They found the following results: (1) touching an aluminum plate increased blood pressure, but the increase was inhibited when the aluminum was warmed; (2) touching an acrylic plastic plate increased blood pressure, with a greater rate of increase in blood pressure when the acrylic plastic plate was chilled; and (3) blood pressure did not change in response to touching objects made of Japanese cypress, Japanese cedar, or oak, and did not increase even when the oak material was chilled. Those reports are pioneering studies on the physiological effects of tactile stimulation with wood on humans; however, there are limitations in that they only used blood pressure, which is an index of autonomic nervous activity measurement of physiological responses.

In this study, we investigated the physiological effects of touching wood in comparison with touching other materials on the left and right prefrontal cortex activity, assessed using near-infrared time-resolved spectroscopy (TRS), and on the autonomic nervous activity, assessed using heart rate variability (HRV).

## 2. Materials and Methods

### 2.1. Participants

The study participants were 18 female university students (mean age, 21.7 ± 1.6 years). We excluded smokers, those currently in treatment for disease, and those with menstrual period during the study period. All participants were informed about the aim of the experiment and the procedures involved in it, and they provided written informed consent to participate. This study was performed in accordance with the regulations of the Ethics Committee of the Center for Environment, Health and Field Sciences, Chiba University, Japan (Project identification code number: 5).

### 2.2. Study Protocol

**Figure 1:**
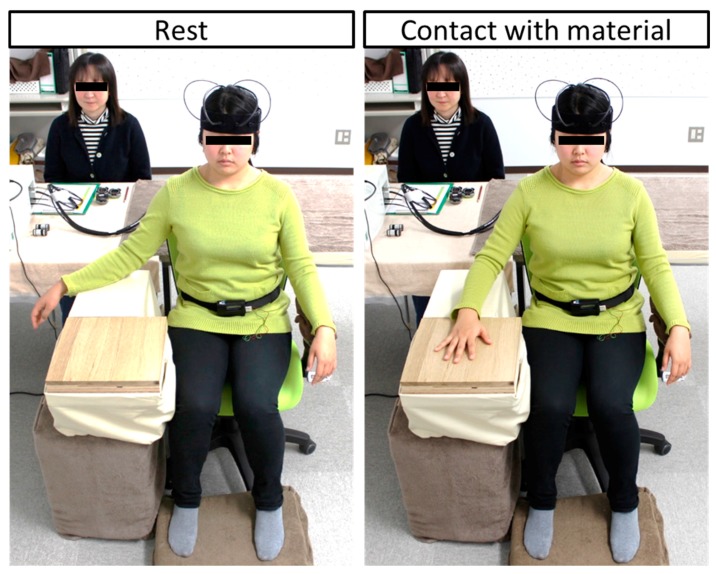
Experimental scene.

**Figure 2:**
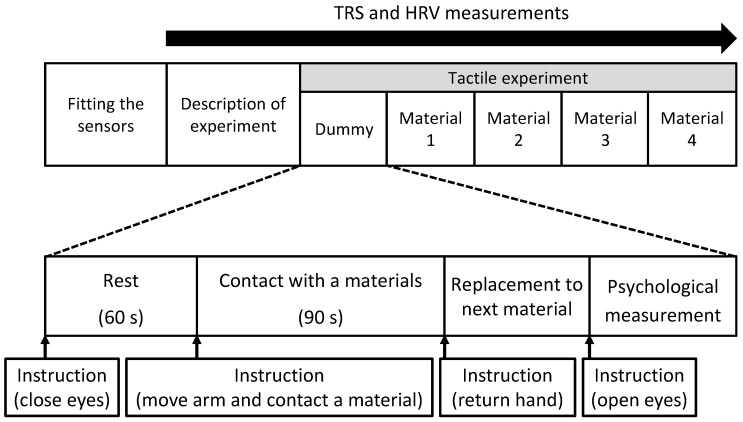
Experimental schedule.

Physiological measurements were performed in a chamber with an artificial climate in the Center for Environment, Health and Field Sciences, Chiba University. This chamber was maintained at 25 °C, 50% relative humidity, and 230-lux illumination. In the waiting room, the participants received a description of the experiment and then moved into the chamber with an artificial climate. After sensors for physiological measurement were fit, participants received a description of the measurement procedure while sitting. After that, they practiced touching a material with their palm using a dummy sample (sheet flooring). The procedure was as follows. Participants rested with their eyes closed for 60 s (Figure 1 left). When receiving instructions from an experimenter, they moved their right forearm using their elbow as a fulcrum, and placed the palm on the material for 90 s (Figure 1 right). After touching the material for 90 s, they returned the hand to the previous position upon instruction of an experimenter (Figure 1 left). The experimenter placed the next material, hid the material with a cloth, and then instructed participants to open their eyes. Subsequently, the participants answered the subjective evaluation test. Figure 2 shows the experimental schedule. Materials were presented in a counterbalanced order to eliminate any effects due to the order of tactile stimulation. The physiological responses were measured continually.

### 2.3. Tactile Stimulation

**Figure 3:**
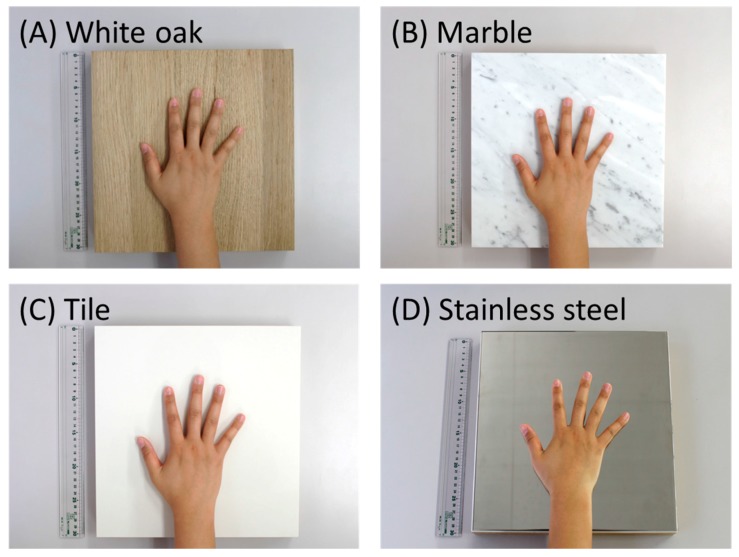
Materials used for the tactile experiment. (**A**) White oak; (**B**) Marble; (**C**) Tile; and (**D**) Stainless steel.

The wood type used was white oak (*Quercus alba*; Figure 3A). Five laminae without vertical joining (the size of one lamina was 300 × 60 × 15 mm) were mutually bonded along the width. To prevent bending, a second bonding was performed using Japanese cedar plywood (300 × 300 × 28 mm), and the thickness of the material was 43 mm. The surface touched by palm was brushed and non-coated. Hereinafter, this is referred to as “white oak”.

As comparable materials, marble (Figure 3B) and ceramic tiles (Figure 3C), which are used as building material, were selected. In addition, stainless steel (Figure 3D) was used as one of the representative artificial materials. The size of all slabs was 300 × 300 mm. The thicknesses of the marble, tile, and stainless steel were 15 mm, 8 mm, and 5 mm, respectively. To render the thickness of all materials presented to the participants uniform at 43 mm, Japanese cedar plywood was adhered under each material. The surfaces of the marble, tile, and stainless steel were processed by buffing. In addition, wax was applied to the tile.

All materials were kept at room temperature. The physical properties of the materials are shown in Table 1.

### 2.4. Physiological Measurement

#### 2.4.1. Near-Infrared Time-Resolved Spectroscopy

As an indicator of brain activity, TRS, which is a near-infrared spectroscopy method, was used. The sensors were mounted at approximately Fp1 and Fp2 of the international 10-20 system (EEG) on the subject’s forehead, and oxyhemoglobin (oxy-Hb) and deoxyhemoglobin (deoxy-Hb) concentrations in the prefrontal cortex were measured (TRS-20 system; Hamamatsu Photonics K.K., Shizuoka, Japan) [10,11,12]. In previous studies [13,14], it has been demonstrated that nature-derived stimuli increased subjective feelings of relaxation and decreased oxy-Hb concentrations in the prefrontal cortex, indicating that these stimuli calmed prefrontal cortex activity. The oxy-Hb concentrations in the left and right prefrontal cortex were measured before the materials were touched (premeasurement condition) and during the 90 s of touching the materials (postmeasurement condition). The data measured by TRS-20 differ in sampling time for all data. In the present experiment, these data were measured at 1.07 to 1.16-s intervals. We transformed the data by linear interpolation every 1 s in order to show the time series data for oxy-Hb concentration in the left/right prefrontal cortex over a 90-s period. In addition, all data were calculated as the difference relative to a 10-s baseline period immediately before participants touched the test material.

#### 2.4.2. Heart Rate Variability

As an indicator of autonomic nervous activity, HRV was analyzed for the periods between consecutive R waves (R–R intervals) on electrocardiograms measured with a portable electrocardiograph (Activtracer AC-301A; GMS, Tokyo, Japan) [15,16]. The power levels of the low-frequency (LF: 0.04–0.15 Hz) and high-frequency (HF: 0.15–0.40 Hz) components of HRV were calculated using the maximum-entropy method (MemCalc/Win; GMS, Tokyo, Japan). The HF power reflected the parasympathetic nervous activity. The LF/HF ratio reflected sympathetic nervous activity [17,18]. To normalize HRV parameters across the participants, we used natural logarithmic transformed values for the analysis [19]. The values of ln(HF) and ln(LF/HF) were acquired changes in each 30 s and overall mean during the 90 s of touching the samples, respectively. In addition, all data were calculated as the difference relative to a 10-s baseline right before touching began.

### 2.5. Psychological Measurement

The modified semantic differential (SD) method [20] and the Profile of Mood State (POMS) [21,22,23,24,25] were used to evaluate the psychological effects of touching the materials. The SD method tests the subjective evaluations of participants through a questionnaire with opposing adjectives, each of which was evaluated on a 13-point scale. Six pairs of adjectives were assessed as “comfortable–uncomfortable”, “natural–artificial”, “relaxed–awakening”, “warm–cold”, “uneven–flat”, and “dry–moist.” The POMS scores were determined for the following six subscales: “tension–anxiety (T–A)”, “depression (D)”, “anger–hostility (A–H)”, “fatigue (F)”, “confusion (C)”, and “vigor (V)” [21,22,23]. The “total mood disturbance (TMD)” score is calculated by the formula [(T–A) + (D) + (A–H) + (F) + (C) – (V)] [24]. A lower score of TMD indicates a better emotional condition [25]. We used a short version of the POMS that included 30 questions to decrease the participants’ burden [23].

### 2.6. Statistical Analysis

Statistical Package for Social Sciences software (v21.0, IBM Corp., Armonk, NY, USA) was used for all statistical analyses. A paired *t*-test with Holm correction was used to compare physiological responses to white oak and the other material (marble, tile and stainless steel). The Wilcoxon signed-rank test with Holm correction was applied to analyze the differences in psychological indices between white oak and the other materials (marble, tile and stainless steel). In all cases, the significance level was set at *p* < 0.05. One-sided tests were used for both comparisons because our hypothesis was that humans would be more relaxed after touching the wood than after touching the other materials.

## 3. Results

### 3.1. Physiological Effects

#### 3.1.1. TRS

**Figure 4:**
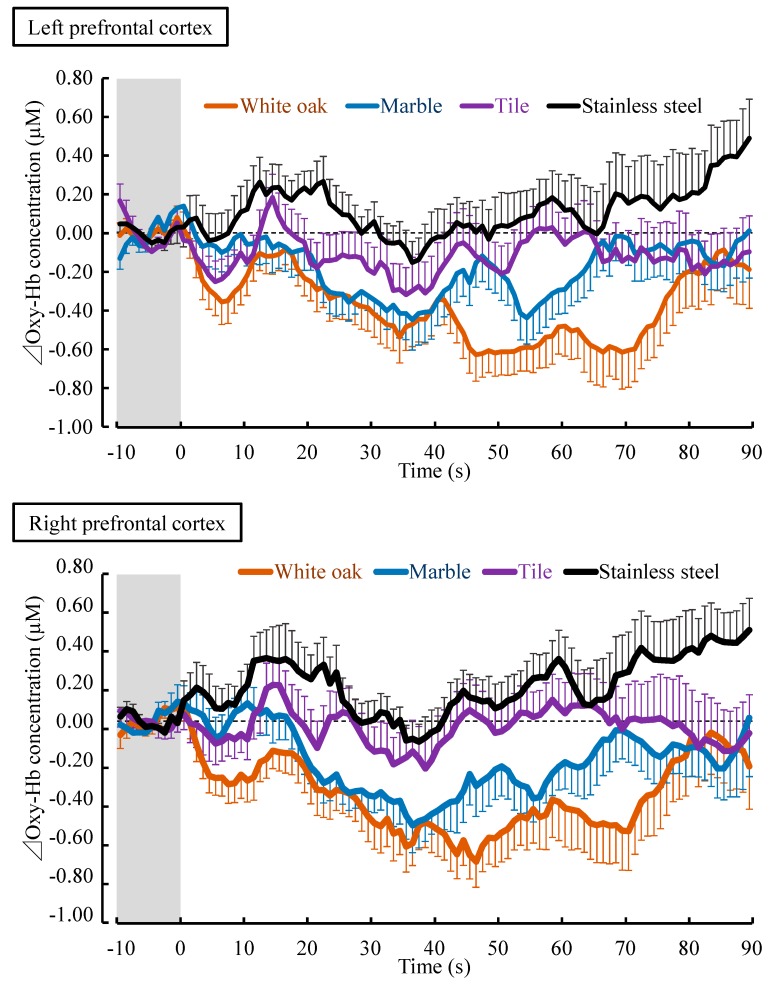
Changes in every 1 s over 90 s oxy-Hb concentration in the left/right prefrontal cortex while touching white oak and other materials (marble, tile, and stainless steel). All data were calculated as the difference relative to a 10-s baseline period immediately before participants touched the test material. Data are expressed as the mean  ±  standard error, *n* = 18.

Figure 4 shows the changes in the oxy-Hb concentration per second in the left/right prefrontal cortex while touching white oak and other materials. The mean baseline for the 10 s oxy-Hb concentration before touching in the left prefrontal cortex did not significantly differ among the four materials (white oak: 43.61 ± 0.81 µM (mean ± standard error), marble: 43.44 ± 0.87 µM, tile: 43.24 ± 0.87 µM, stainless steel: 43.03 ± 0.83 µM; *p* > 0.05). There was also no significant difference in the baseline 10-s oxy-Hb concentration of the right prefrontal cortex (white oak: 43.29 ± 1.10 µM, marble: 42.97 ± 1.15 µM, tile: 43.32 ± 1.11 µM, stainless steel: 43.21 ± 1.09 µM; *p* > 0.05).

The oxy-Hb concentrations in the left/right prefrontal cortex immediately decreased after touching white oak with the palm and remained lower than the value before touching until the end of contact. With stainless steel, oxy-Hb concentrations gradually increased during the contact. With marble and tiles, they showed a change between the white oak and stainless steel.

**Figure 5:**
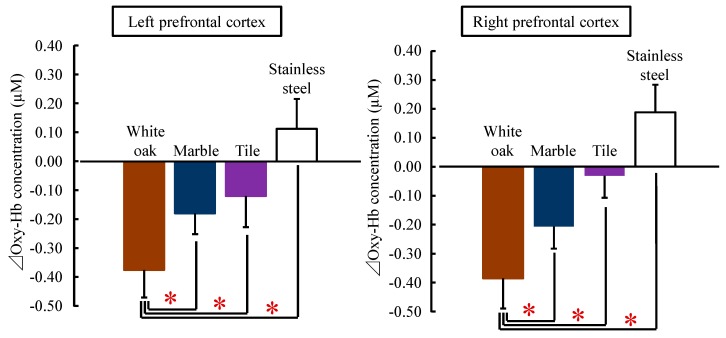
The overall mean oxy-Hb concentrations in the right and left prefrontal cortex while touching white oak and other materials (marble, tile, and stainless steel). Data are expressed as the mean ± standard error. *n* = 18, * *p* < 0.05 as determined by the paired *t*-test; Holm correction was applied.

The comparison of the overall mean oxy-Hb concentration in the left/right prefrontal cortex while touching white oak and other materials is shown in Figure 5. Touching white oak significantly decreased the oxy-Hb concentration in the left prefrontal cortex compared with marble, tile, and stainless steel (white oak: −0.37 ± 0.10 µM, marble: −0.18 ± 0.07 µM, tile: −0.12 ± 0.11 µM, stainless steel: 0.11 ± 0.10 µM; white oak vs. marble: t(17) = −2.283, *p* = 0.018; white oak vs. tile: t(17) = −1.929, *p* = 0.032; white oak vs. stainless steel: t(17) = −4.242, *p* < 0.001; Figure 5 left). Similarly, in the right prefrontal cortex, the mean oxy-Hb concentration while touching white oak was −0.38 ± 0.10 µM, which was significantly lower than that while touching other materials (marble: −0.21 ± 0.08 µM, tile: −0.03 ± 0.11 µM, stainless steel: 0.19 ± 0.09 µM; white oak vs. marble: t(17) = −1.985, *p* = 0.032; white oak vs. tile: t(17) = −3.017, *p* = 0.004; white oak vs. stainless steel: t(17) = −8.341, *p* < 0.001; Figure 5 right).

However, there was no significant difference in deoxy-Hb concentration in the left/right prefrontal cortex when participants touched white oak vs. other materials (marble, tile, and stainless steel).

#### 3.1.2. HRV

**Figure 6:**
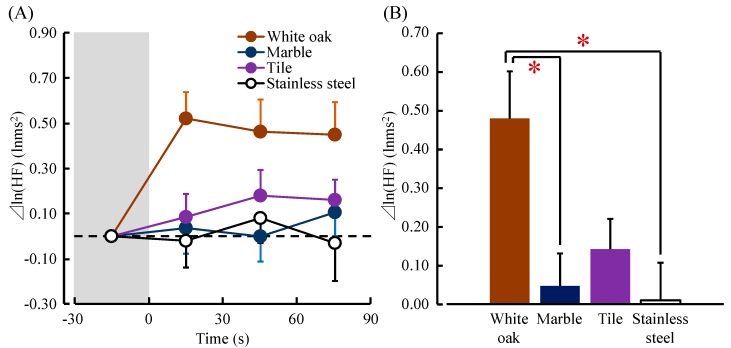
The 30 s averages and overall mean of the natural logarithm of the high-frequency component (HF) of heart rate variability (HRV) while touching white oak and other materials (marble, tile, and stainless steel). (**A**) Changes in each 30 s average HF value over 90 s. (**B**) Overall mean HF values. Data are expressed as the mean ± standard error, *n* = 18, * *p* < 0.05 as determined by the paired *t-*test; Holm correction was applied.

Figure 6A shows the changes in the ln(HF) value, which reflected parasympathetic nervous activity while touching white oak and other materials. The mean baseline value of ln(HF) at 30 s before touching did not significantly differ among the four materials (white oak: 5.56 ± 0.18 lnms^2^, marble: 5.67 ± 0.19 lnms^2^, tile: 5.56 ± 0.19 lnms^2^, stainless steel: 5.61 ± 0.21 lnms^2^; *p* > 0.05).

The ln(HF) value immediately increased after contact with white oak, and it remained higher than the value before touching until the end of the contact. However, the changes in the ln(HF) value during contact with other materials (marble, tile and stainless steel) were small.

Figure 6B shows the overall mean of the ln(HF) value when touching white oak and other materials. Touching white oak significantly increased the ln(HF) value compared to touching marble and stainless steel (white oak: 0.48 ± 0.12 lnms^2^, marble: 0.05 ± 0.08 lnms^2^, stainless steel: 0.01 ± 0.10 lnms^2^; white oak vs. marble: t(17) = 2.854, *p* = 0.005; white oak vs. stainless steel: t(17) = 3.415, *p* = 0.002; Figure 6B). However, there was no significant difference in the ln(LF/HF), which is an index of sympathetic nervous activity between white oak and the other materials (white oak: −0.84 ± 0.24, marble: −0.47 ± 0.21, tile: −0.14 ± 0.25, stainless steel: 0.08 ± 0.22; *p* > 0.05).

### 3.2. Psychological Effects

**Figure 7:**
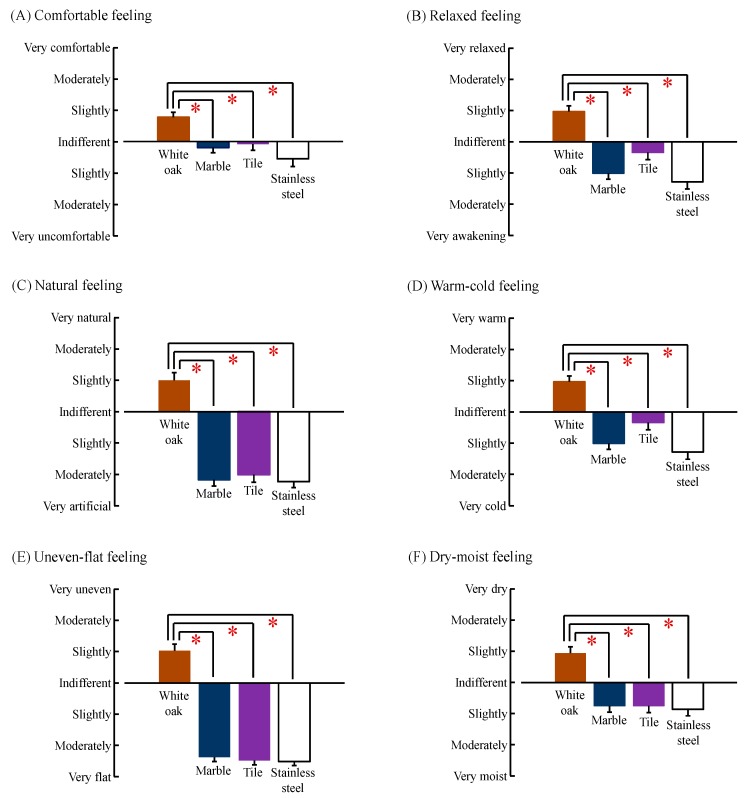
Subjective feeling measured by the modified semantic differential method after touching white oak and other materials (marble, tile, and stainless steel). (**A**) Comfortable feeling; (**B**) Relaxed feeling; (**C**) Natural feeling; (**D**) Warm–cold feeling; (**E**) Uneven–flat feeling; and (**F**) Dry–moist feeling. Data are expressed as the mean ± standard error, *n* = 18, * *p* < 0.05 as determined by the Wilcoxon signed-rank test; Holm correction was applied.

The results of subjective evaluation by the modified SD method are shown in Figure 7. In terms of the “comfortable feeling”, participants provided subjective reports of feeling “slightly comfortable” after contact with white oak; however, they provided reports of feeling “indifferent to slightly uncomfortable” after touching other materials. Therefore, touching the white oak was believed to induce significantly more comfort than touching other materials (white oak vs. marble: *p* = 0.001; white oak vs. tile: *p* = 0.002; white oak vs. stainless steel: *p* = 0.001; Figure 7A). Also in the “relaxation feeling”, subjects reported feeling “slightly relaxed” while touching white oak; however, they reported feeling “indifferent to slight awakening” while touching a tile and “slight to moderate awakening” while touching marble and stainless steel. Thus, white oak induced significantly more relaxation than other materials (white oak vs. marble: *p* < 0.001; white oak vs. tile: *p* = 0.001; white oak vs. stainless steel: *p* < 0.001; Figure 7B). Regarding the “natural feeling”, white oak, which was perceived as “slightly natural”, was considered significantly more natural than the other materials, which were perceived as “moderately to very artificial” (white oak vs. marble: *p* < 0.001; white oak vs. tile: *p* < 0.001; white oak vs. stainless steel: *p* < 0.001; Figure 7C). Furthermore, in “warm–cold”, “uneven–flat”, and “dry–moist” feelings while touching the white oak, significantly higher scores were obtained for “warm”, “uneven”, and “dry” compared with scores for other materials (white oak vs. marble: *p* < 0.001; white oak vs. tile: *p <* 0.001; white oak vs. stainless steel: *p* < 0.001; Figure 7D; white oak vs. marble: *p <* 0.001; white oak vs. tile: *p* < 0.001; white oak vs. stainless steel: *p* < 0.001; Figure 7E; white oak vs. marble: *p* < 0.001; white oak vs. tile: *p* = 0.001; white oak vs. stainless steel: *p* < 0.001; Figure 7F).

**Figure 8:**
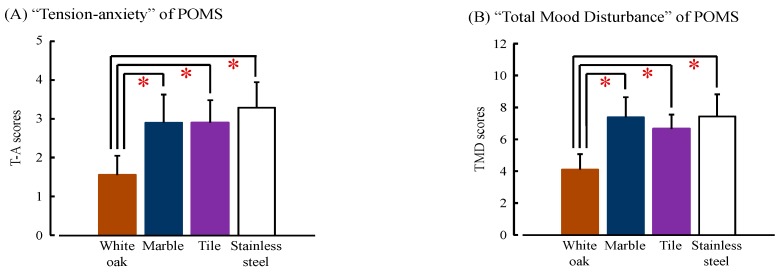
“Tension–anxiety (T–A)” and “total mood disturbance (TMD)” score on the Profile of Mood State (POMS) test after touching white oak and other materials (marble, tile and stainless steel). (**A**) T–A scores of POMS; and (**B**) TMD scores of POMS. Data are expressed as the mean ± standard error, *n* = 18, * *p* < 0.05 as determined by the Wilcoxon signed-rank test; Holm correction was applied.

The mood state in the short version of the Profile of Mood State (POMS) scale is shown in Figure 8. The score for the negative subscale “tension–anxiety” was significantly lower after touching white oak than that after touching other materials (white oak vs. marble: *p* = 0.006; white oak vs. tile: *p* = 0.001; white oak vs. stainless steel: *p* = 0.001; Figure 8A). Furthermore, the score for “total mood disturbance” was significantly lower after touching white oak than that after touching other materials (white oak vs. marble: *p* = 0.001; white oak vs. tile: *p* < 0.001; white oak vs. stainless steel: *p* = 0.001; Figure 8B). For the other subscales (“depression”, “anger−hostility”, “fatigue”, “confusion”, and “vigor”), no significant differences were observed.

## 4. Discussion

This study aimed to clarify the effects of touching wood in comparison with touching other materials on the activity in the left and right prefrontal cortex, assessed using TRS, and on autonomic nervous activity, assessed using HRV. The results showed that in comparison with touching other materials, touching white oak significantly decreased the oxy-Hb concentration in the right prefrontal cortex and significantly increased parasympathetic nervous activity according to the 90-s overall mean values.

Previous studies on the physiological effects brought about by forests, which are a typical natural environment, have demonstrated that looking at forests can decrease total oxy-Hb concentration in the prefrontal cortex [26], increase parasympathetic nervous activity [27,28,29,30], and decrease heart rate or pulse rate [27,31,32] compared with observing an urban area. Our findings concentrated on the tactile stimulating effect of wood are consistent with those of previous studies.

In recent years, several studies have been performed on the physiological effects caused by olfactory stimulation using wood. The effect on prefrontal cortex activity of olfactory stimulation by wood chips of Japanese cypress, a coniferous tree, treated with different drying methods has been reported [33]. The physiological effects of olfactory stimulation with “air-dried wood”, which was produced through natural drying processes over 45 months, and with “high-temperature-dried wood”, which was produced using steam heating drying equipment at a high temperature and high speed, were compared. Olfactory stimulation using air-dried wood of Japanese cypress decreased the oxy-Hb concentration in the prefrontal cortex more than high-temperature-dried wood did [33]. The effects of olfactory stimulation with Japanese cypress leaf oil on the brain activity and autonomic nervous activity have also been investigated [34]. Olfactory stimulation with Japanese cypress leaf oil induced a reduction in oxy-Hb concentration in the right prefrontal cortex and increased parasympathetic nervous activity (the HF power of HRV) compared to the control condition (air), indicating that olfactory stimulation with Japanese cypress leaf oil can induce physiological relaxation [34]. Inhalation of α-pinene, which is a major odor component contained in Japanese cedar and Japanese cypress, increased parasympathetic nervous activity and decreased heart rate compared with control (air), indicating physiological relaxation [35]. Similarly, inhalation of D-limonene, one of the main volatile components of wood, increased HF power and decreased the heart rate compared with the control condition (air), suggesting that D-limonene induced physiological relaxation [36]. Regarding the influence of touching some kinds of wood with the palm of the hand, Sakuragawa et al. [7] examined differences in the effects of tactile stimulation on blood pressure. Participants touched the surface of each material for 60 s with their eyes closed. This produced the following results: (1) blood pressure rose transiently just after touching Japanese cypress, Japanese cedar, and oak, but the change did not persist; and (2) blood pressure was at a high value even after the transient rise while touching artificial materials (aluminum or acrylic plastic plate).

To explain this phenomenon, Miyazaki has advocated a “back to nature” theory [37,38]. In this theory, they claim human physiological functions are best adapted to a natural environment because over 99.99% of the course of human evolution since our ancestors started evolving from a subset of primates into our current form around 6–7 million years ago has been spent in a natural environment. Conversely, we have spent less than 0.01% of our species’ history in a modern environment, which only started with the beginning of urbanization in the Industrial Revolution. The highly urbanized and artificial environments that we currently inhabit are the root cause of the “stress state” in modern people. We therefore enter a relaxed state when exposed to the natural environment or to a nature-derived stimulation, which brings us closer to our original natural state as human beings. Tactile stimulation by touching wood with the palm is considered to bring about physiological relaxation effects because wood is a familiar and representative natural material for humans.

Regarding subjective evaluations, the participants felt more comfortable, relaxed, natural, warm, uneven, and dry after contact with white oak than with other materials. In this study, the physiological response of brain activity and autonomic nervous activity, the subjective evaluation of materials, and evaluations of their physical properties (e.g., surface roughness and heat flow rate) are shown to be consistent. The relationship between the subjective evaluation when touching wood and the physical property values of materials has been examined for a long time. In particular, there are many previous studies focusing on subjective thermal sensation and thermal properties of materials. Wang et al. [39] reported a significant correlation between the subjective thermal sensation and the heat penetration coefficient of materials. Sadoh and Nakato [40] clarified that the thermal conductivity of wood and the heat flux between wood and palm affect the subjective thermal sensation. Sakuragawa et al. [41] reported a significant correlation between heat flux between materials and the palm and the subjective thermal-comfort feeling using wood and other materials, such as aluminum. In addition, equality of the heat flux value at the base of the palm and the heat flux value between wood and palm is cited as a factor that increases subjective comfort upon touching wood with the palm compared to other materials. Furthermore, Sakuragawa et al. [7] examined the physiological effects of tactile stimulation of the palm with wood on blood pressure, using materials at different temperatures (cool, room temperature, and warm) to eliminate the influence of the heat flux of each material. As a result, systolic blood pressure did not increase even upon contact with chilled wood, and the subjective feeling of “coarse/natural” was maintained. Conversely, touching aluminum at room temperature increased blood pressure, and subjective feelings of “dangerous/uncomfortable” and “flat/artificial” were increased. These results revealed that even in a cooled state, touching wood did not cause physiological stress.

In this study, we examined the physiological relaxation effect of touching wood in comparison with touching other materials with the palm of the hand. However, this study had three limitations. First, although this study used non-coated wood, it is necessary to clarify the influence of touching wood with various coatings on the physiological response because much of the wood used in everyday life is coated. Second, although we clarified the effect of touching wood with the palm here, the effect when touching with the sole of the foot should also be examined because wood is often used as flooring material. Third, this study measured the physiological effects of only placing the palm on the material. The influence of active contact, such as stroking the surface of the wood with the hand, on the physiological response should also be clarified.

## 5. Conclusions

In comparison with other materials (marble, tile and stainless steel), tactile stimulation of the palm with white oak significantly decreased the oxy-Hb concentration in the left/right prefrontal cortex, which is associated with prefrontal cortex activity, and significantly increased the ln(HF) component of HRV, which reflected parasympathetic nervous activity. These findings indicate that contact with wood induces physiological relaxation.

## Figures and Tables

**Table 1:** Details of materials.

Material	*h* (mm)	λ (W/(m-K)) ^1^	*R* (µm) ^2^	Conditioning
White oak	15 (+JCP 28)	0.120	57.10	Brushing
Marble	15 (+JCP 28)	0.146	0.09	Buffing
Tile	8 (+JCP 35)	0.144	0.09	Buffing with wax coating
Stainless steel	5 (+JCP 38)	0.336	0.02	Buffing

^1^ A heat flow meter (HFM 436 Lambda; NETZSCH, Selb, Germany) tuned according to ASTM C518-10 [8] and ISO8310 [9], was used. The direction of heat flow was vertically downward. The temperatures of the high- and low-temperature heat plates were 35 °C and 15 °C, respectively. The thermal conductivity at an average material temperature of 25 °C was calculated. The test specimens were used with the cedar plywood attached; ^2^ A contact surface roughness profilometer (SE3500; Kosaka Laboratory Ltd., Tokyo, Japan) with a diamond needle was used. The evaluation length was 50 mm. The central portion of the samples was measured five times with a 50 mm spacing, and the average value was calculated; *h*: Thickness of material; *λ*: thermal conductivity; *Ra*: arithmetic average roughness; *JCP*: Japanese cedar plywood.
